# Shrimp Consumption after *Deepwater Horizon:* No Evidence of Excess Risks for Vietnamese Americans

**DOI:** 10.1289/ehp.123-A45

**Published:** 2015-02-01

**Authors:** Janet L. Pelley

**Affiliations:** Janet L. Pelley, MS, based in Toronto, ON, Canada, writes for *Chemical & Engineering News* and *Frontiers in Ecology and the Environment*.

After the *Deepwater Horizon* oil well blowout on 20 April 2010, fisheries in the Gulf of Mexico were closed while officials screened seafood for unsafe levels of hydrocarbon contaminants.^1^ But when federal agencies began reopening fisheries in the summer of 2010, the U.S. Food and Drug Administration’s (FDA) affirmation of seafood safety was met with distrust, in part because some felt the agency’s risk assessment didn’t account for coastal populations who eat a heavy diet of seafood.^2^ A health risk assessment reported in *EHP* targets one such group—Vietnamese-Americans in southeast Louisiana who ate locally caught shrimp after the oil spill. The results support the FDA’s estimates, suggesting no acute health effects or excess cancer risk among this population.^3^

In its initial risk assessment the FDA calculated levels of concern in seafood for 13 high-priority polycyclic aromatic hydrocarbons (PAHs) and their related chemical variations.^4^ The FDA assumed an average consumer weight of 80 kg (176 lbs) and daily average shrimp/crab consumption of 13 g (about 0.5 oz), reflecting the intake of the 90th percentile of U.S. seafood eaters as reported by the National Health and Nutrition Examination Survey (NHANES).^5^

**Figure:**
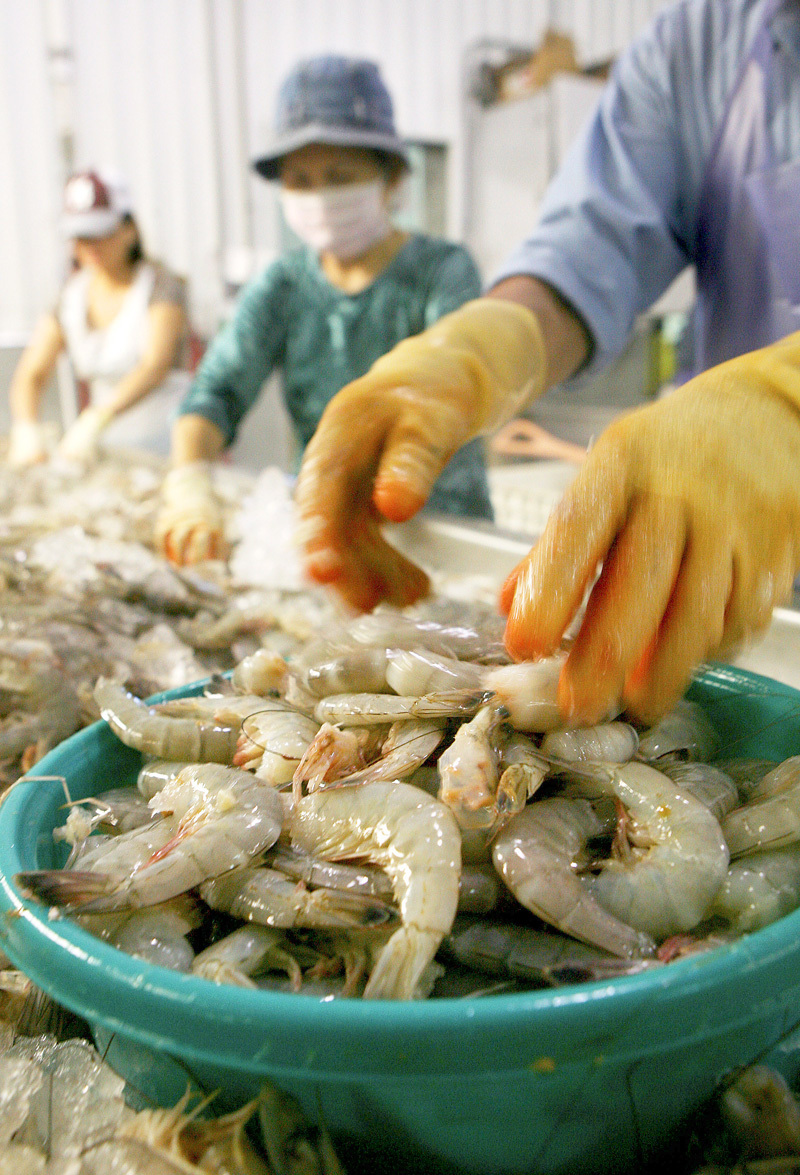
From the standpoint of both economics and diet, shrimp plays a key role in Vietnamese-American communities living along the U.S. Gulf Coast. © Sean Gardner/Reuters

But those assumptions left out the highest 10% of seafood consumers, among them Vietnamese Americans living along the Gulf Coast. “The Vietnamese-American population in eastern New Orleans, Louisiana, is a worst-case scenario for risk because they eat more shrimp and weigh less than the average citizen,” says study leader Mark Wilson, an environmental toxicologist at Tulane University. Indeed, in contrast to the FDA assumptions, the individuals in this study had an average body weight of 63 kg (139 lbs) and consumed an average of 45.2 g (1.6 oz) of shrimp daily.^3^

Wilson and his colleagues involved members of the Vietnamese-American community in their study, following a community-based participatory research model.^6^ Seven months after the blowout the investigators collected samples of white shrimp (*Litopenaeus setiferus*) from oiled and non-oiled fishing areas used by community members. Using gas chromatography/mass spectrometry, they assessed shrimp samples for 81 PAHs and detected 8 at levels comparable to those reported in other studies, including the FDA’s risk assessment. The remaining 73 PAHs were below the limit of detection.^3^

In spring 2012 the investigators surveyed 115 Vietnamese-American men and women, collecting information on shrimp consumption before, during, and after the spill. The team ran Monte Carlo simulations on the data to estimate health risks based on the body weights and daily shrimp consumption of study participants, concentrations of contaminants measured in the shrimp, and estimated risks for each contaminant. For noncancer health effects, even the highest estimated level of PAH exposure from shrimp was about 10,000 times less than the reference dose defined by the U.S. Environmental Protection Agency (EPA).^3^

Of the 81 PAHs that were evaluated, 7 are classified as carcinogenic. None of these 7 were present at levels above the minimum detection limit for the assays used. But because nondetected chemicals may be present at concentrations too low to be positively identified by the assay, the authors estimated risks anyway, using a common substitution method employed in risk assessment. The resulting estimated risk for consumption of the 7 carcinogenic PAHs was 10 times lower than the EPA’s acceptable level of risk of 1 case in 1 million. When the investigators factored in the 8 PAHs that actually were detected in shrimp, the risk level increased to roughly equal to the maximum acceptable level set by the FDA—1 in 100,000. In a third iteration, they included the chemicals that were actually detected plus assumed the presence of all of the other analytes, including the 7 carcinogenic PAHs. In this iteration the calculated cancer risk from eating local shrimp increased to as high as 1 in 10,000.^3^

Wilson emphasizes that this apparent increase in risk is not based on chemicals that were actually detected. “They may be there or they may not be, and we choose to assume that they were present,” he says. For those chemicals with established regulatory limits, Wilson says the results indicate that PAH contamination of shrimp after the oil spill was not a serious health concern for this sensitive population.

One weakness of the study is that it included no children and very few pregnant women, groups who are most at risk from contaminants, points out Miriam Rotkin-Ellman of the Natural Resources Defense Council, who coauthored a 2012 critique of the FDA risk assessment.^2^ She says the study points to the need for the FDA to set levels of concern for more PAHs and consider vulnerable subpopulations in risk assessments, as recommended by the National Academy of Sciences.^7^

The authors concur with the need for a more inclusive approach, writing, “Finding no unacceptable risk among [vulnerable] groups would suggest that the rest of the general population will also have no unacceptable health risks … [and] mitigate the criticism that federal policy-based risk assessment practices ignore the safety of sensitive subpopulations.”^3^
